# Impact of Chemotherapy on Diet and Nutritional Status of Women with Breast Cancer: A Prospective Study

**DOI:** 10.1371/journal.pone.0157113

**Published:** 2016-06-16

**Authors:** Isis Danyelle Dias Custódio, Eduarda da Costa Marinho, Cristiana Araújo Gontijo, Taísa Sabrina Silva Pereira, Carlos Eduardo Paiva, Yara Cristina de Paiva Maia

**Affiliations:** 1 Graduate Program in Health Sciences, Federal University of Uberlandia, Uberlandia, Minas Gerais, Brazil; 2 Graduate Program in Public Health, Federal University of Espírito Santo, Vitória, Espírito Santo, Brazil; 3 Department of Clinical Oncology, Graduate Program in Oncology, Palliative Care and Quality of Life Research Group (GPQual), Pio XII Foundation—Barretos Cancer Hospital, Barretos, Sao Paulo, Brazil; University of South Alabama Mitchell Cancer Institute, UNITED STATES

## Abstract

Certain food groups are often rejected during chemotherapy (CT) due to the side effects of treatment, which may interfere with adequate diet and nutritional status. The aim of this study was to evaluate the treatment impact on the diet and nutritional status of women with breast cancer (BC). In this prospective longitudinal study, conducted in 2014–2015, 55 women diagnosed with BC, with a mean age 51.5±10.1 years, were followed and data were collected at three different times. Anthropometric and dietary assessments were performed, the latter by applying nine 24h dietary recalls, by using the Brazilian Healthy Eating Index Revised (BHEI-R), and calculating the prevalence of inadequacy by the EAR cut-off point method. Regarding the BHEI-R analysis, the majority of women had a “diet requires modification’, both at the beginning (T0, 58.2%, n = 32) and during treatment (T1, 54.5%, n = 30). However, after the end of the CT, the greater percentage of patients (T2, 49.1%, n = 27) were classified as having an "inadequate diet", since the Total Fruit consumption as well as the Dark Green and Orange Vegetable and Legume consumption decreased significantly during treatment (p = 0.043 and p = 0.026, respectively). There was a significant reduction in the intake of macro and micronutrients, with a high prevalence of inadequacy, of up to 100%, for calcium, iron, phosphorus, magnesium, niacin, riboflavin, thiamin, vitamin B6, vitamin C and zinc. Assessment of the nutritional status indicated that 56% (n = 31) of patients were overweight at these three different times. Weight, BMI and Waist Circumference increased significantly, indicating a worse nutritional status, and there was a correlation between poor diet quality and higher values for BMI, Waist-Hip Ratio and Waist-to-Height Ratio. Chemotherapy interferes in the patients’ diet generating a negative impact on the quality and intake of micro and macronutrients, as well as an impact on their nutritional status, with an increase in anthropometric measurements.

## Introduction

The number of cases and annual deaths from cancer by 2035 is expected to be 23.9 and 14.6 million, respectively, representing an increase of 69.5% in the number of cases, and 78% in the number of deaths compared to 2012 [1]. The number of new cancer cases registered in 2012 was 14.1 million (excluding non-melanoma skin cancers) and, of this total, 11.8% were from breast cancer, the second most common type which is just behind cancer lung (12.9%) [2]. The reasons for that increase are related to population growth and increased longevity [1].

In Brazil, it is estimated that by 2016 approximately 596,000 cases of cancer will be registered, and of these, approximately 58,000 will be new cases of breast cancer, the predominant type among women in the country [3].

Modifiable behaviors, like improvements in the quantity and quality of food consumed may contribute to healing, recovery and breast cancer survival [4]. The adoption of healthy eating habits with a high consumption of fruits, vegetables, whole grains, poultry and fish, instead of a western dietary pattern characterized by a high consumption of refined grains, sweets, high-fat dairy products, and red and processed meats, might improve the overall prognosis and survival of women diagnosed with early-stage breast cancer [5].

Studies evaluating changes in eating patterns after the diagnosis of breast cancer showed different results, but most found favorable changes with a reduced consumption of high-fat and high-sugar foods, and an increase in vegetables and fruits [6, 7]. Certain food groups, are often rejected or preferred during chemotherapy [8–10], because of side effects of treatment, such as nausea and vomiting [11]. These changes in diet can interfere with an adequate diet and influence the nutritional status of the patient, and there may be consequences for the prognosis of the disease. For these patients, changes in dietary patterns are not always accompanied by weight reduction, since 50–96% of women with breast cancer have weight gain during treatment, with progressive gains in the months and years following the diagnosis [12, 13].

The initial excess weight, or acquired during the development of the disease, is a factor which negatively influences the prognosis, quality of life and survival of women affected by breast cancer [14, 15], in part due to alterations in the production of cytokines and inflammation [16, 17]. Moreover, even without weight gain, these women are affected by adverse changes in body composition, with frequent sarcopenia (loss of muscle mass), accompanied by fat gain, which represents a significant risk for the development of comorbidities and long term survival [12, 18].

The overall diet pattern can be evaluated with the use of dietary indexes relating the diet quality to the prognosis [19] and to the risk of death from breast cancer [20–22]. Among the indexes that assess diet quality, there is the Healthy Eating Index (HEI) [23], which was adapted and validated for the Brazilian population, resulting in the Brazilian Healthy Eating Index (BHEI) [24]. With the review and update of the HEI [25] and publication of the Food Guide for the Brazilian Population [26], the BHEI was adapted to suit the new recommendations, becoming the Brazilian Healthy Eating Index Revised (BHEI-R) [27]. This index is formed by a twelve-component system that determines various aspects of a healthy diet regarding nutritional recommendations [27], having been confirmed as a reliable instrument and structurally valid to assess and monitor the quality of Brazilian diets [28].

There are still gaps in knowledge and a need for more information regarding the side effects of chemotherapy associated with nutrition and healthy eating [29]. So, considering that the adverse effects of chemotherapy are able to cause changes in the diet and nutritional status, detailed knowledge of these changes may contribute to the direction of the guidelines and behaviors aimed at these patients. Furthermore, little is known about intake and inadequacy of micro and macronutrients during treatment.

The main goal of this study was to evaluate the impact of chemotherapy on the diet and nutritional status of women with breast cancer. A secondary goal was to assess the relationship between the nutritional status and diet quality. The hypothesis was that the diet quality, as well as the supply of nutrients, would be adversely affected by chemotherapy, and this change would be associated with a worsening of nutritional status.

## Methods

### Ethical aspects

This study was approved by the Human Research Ethics Committee (protocol number 721.977/14) and the entire study was conducted based on the standards of the Declaration of Helsinki. All participants signed a free and informed consent form.

### Study design and clinical setting

A prospective longitudinal study conducted in 2014–2015, in a Brazilian university hospital (HC-UFU, Uberlandia, Minas Gerais, Brazil) including three sequential assessments with breast cancer patients during chemotherapy. The follow-up time varied according to the chemotherapy regimen, for about 4 to 6 months.

### Sample size calculation

All the eligible women during the time of the study were invited to participate.

The sample size required for this study was determined using the G*Power software, version 3.1 [30]. The sample size calculations were based on an F test ANOVA repeated measures with effect size f of 0.25, an alpha level of 0.05, 95% power, one group of individuals and three measurements. Given these specifications, a total sample of 43 women was required at final follow-up, having been the result of the calculation that required the larger minimum sample. Considering a 20% adjustment for possible losses, a minimum of 52 women was needed at baseline (T0).

### Eligibility criteria

In this study included women aged 18 years or older, diagnosed with breast cancer, who were in the first cycle of chemotherapy and who had the physical, verbal and cognitive ability needed to respond to the tools necessary to data collection.

Exclusion criteria were age below 18 years, primary tumor site other than the breast, anticancer treatment that did not include chemotherapy, not the first cycle of chemotherapy, and physical and/or mental inability to respond to the questionnaires.

### Database

Data collection was performed from August 2014 to October 2015. The volunteers, regardless of the purpose of chemotherapy (curative, neoadjuvant, adjuvant or palliative) and disease stage, were selected while awaiting medical consultation in the waiting room of the cancer center of this hospital.

Assessments were conducted at three different times during the chemotherapy, called T0, period after administration of the first cycle of chemotherapy; T1, period after administration of the intermediate cycle; and T2, period after administration of the last cycle of chemotherapy.

### Analyzed variables

#### Anthropometric assessment

A mechanical scale was used to measure weight, with sensitivity of 100g; for height, a vertical stadiometer with a 1 mm precision scale was used; and for waist circumference (WC) and hip circumference (HC) a flexible and inelastic tape was used, following the protocol recommended by the World Health Organization [31].

After obtaining these measurements, the BMI were calculated dividing weight by height squared (kg/m^2^). Waist-Hip Ratio (WHR) [32] and Waist-to-Height Ratio (WHTR) were also determined [33].

#### Quantitative dietary assessment

Properly trained nutritionists collected information about food consumption by means of a 24-hour dietary recall (24HR) applied through telephone interviews, according to the technique used in the Vigitel Study [34] with adaptations. At each data collection time (T0, T1 and T2), three nonconsecutive 24HR were applied, including a day of the weekend, in order to better reflect the eating habits of the participants, totaling nine dietary questionnaires during the study. Telephone calls were made starting from the day after the infusion of chemotherapy and before the next appointment (Δt = 21 days). However, to avoid the acute effect of chemotherapy, the 24HR were not applied in the first week after the infusion. The 24HR were applied preferably in the second week.

From the 24HR, the mean quantity of total energy, carbohydrate, protein, lipid, dietary fiber, total cholesterol, calcium, iron, phosphorus, magnesium, manganese, niacin, potassium, riboflavin, sodium, thiamin, vitamin B6, vitamin C, zinc, and monounsaturated, polyunsaturated and saturated fats were estimated. Quantification of nutrients was performed through Dietpro® software, version 5.7, using as a reference, preferably, the Brazilian Table of Food Composition [35]. However, for those foods not found in this table, the international reference was used, the table from the United States Department of Agriculture [36].

Due to intrinsic variability of food consumption, values related to energy consumption and nutrients were disattenuated, i.e., adjusted by intra-individual variability to obtain an estimate of individual consumption of energy and nutrients [37], using PC-side software (Department of Statistics, Iowa State University, Iowa, USA). Later, in order to adjust the estimates of nutrients, these were adjusted for the residual method for the total energy of the sample [38], using the SPSS software, version 15.0 for Windows (SPSS Inc., Chicago, Illinois, USA).

From the means and standard deviation, adjusted for energy, of disattenuated nutrients, it was possible to establish the prevalence of inadequacy by the EAR method (Estimated Average Requirement) as the cut-off point [39]. As such, the Z value was calculated ((EAR—Average intake) / standard deviation)) and the Z table curve was consulted to verify the corresponding percentage of individuals with intakes below the EAR. For fiber, manganese, potassium and sodium, which have no EAR value established, the intake comparison was made with their respective AI values (Adequate Intake), and, for those who had an intake above AI, adequacy regarding UL was verified (Tolerable Upper Intake Level).

Distribution of macronutrients in relation to Total Energy Value (TEV) was analyzed using the AMDR (Acceptable Macronutrients Distribution Range) values as a reference [40]. For cholesterol, and monounsaturated, polyunsaturated and saturated fats, the recommendation of the Food and Agriculture Organization of the United Nations was used [41].

#### Qualitative dietary assessment

The qualitative dietary assessment was performed using the Brazilian Healthy Eating Index Revised (BHEI-R), from the following components and, or food groups: Total Fruits (including fruits and natural fruit juices); Whole Fruits (excluding fruit juices); Total Vegetables (including legumes after reaching the maximum score for Meat, Eggs and Beans); Vegetables Dark Green and Orange Vegetables and Legume (including legumes after reaching the maximum score for Meat, Eggs and Beans group, and Total Vegetables); Total Grains (including grains, roots and tubers); Whole Grains; Milk and Dairy Products (including milk and dairy products, as well as soy based drinks); Meat, Eggs and Beans; Oils (including mono and polyunsaturated fats, oilseed oils, and fish fat); Saturated Fat; Sodium; and SoFAAS (calories from solid fats, alcohol and added sugars) [27].

The data with home measurements from the 24HR were converted to units of measurement (grams or milliliters) by Dietpro^®^ software, and then the number of servings and total score and for each Index food group were calculated.

The number of daily servings was adjusted by the energy density. A maximum score (five or ten points) was given to food groups with consumption equal to or greater than the portions recommended by the Food Guide [26], considering 1000 kcal/day; for groups with absent consumption, zero score; and for intermediate values of consumption, a calculation proportional to the amount consumed was performed [27]. For components such as saturated fat, sodium and SoFAAS, the greater the intake, the lower the score assigned.

The maximum score (10 points) for saturated fat was given to percentages of up to 7% of the TEV; a score of 8 points to 10% of TEV; and a score of zero for consumption of this fat above 15% of TEV [27]. For sodium, the maximum score (10 points) was given for consumption of up to 0.75g/ 1000 kcal; a score of 8 points for consumption of up to 1g/ 1000 kcal, with this cut-off point corresponding to the maximum recommended by the Food Guide [26]; and a score of zero for consumption above 2g/ 1000 kcal, corresponding to twice the recommendation [26, 27]. To calculate SoFAAS, calories from solid fats (saturated and *trans* fats), alcohol, and added sugars were summed up, and the percentage in relation to TEV was calculated, with a maximum score (20 points) for consumption at or below 10%, and a score of zero for consumption at or above 35% of TEV [27].

To rate the diet quality of each patient, a stratification into tertiles at the three times (T0, T1 and T2) was performed, considering equivalent ranges of the BHEI-R total scores at baseline (T0), a reference time to identify the treatment impact on acceptance of the different Index components and food groups. Thus, classification was made according to the following cut-off points: "inadequate diet" for scores lower than 63.5; "diet requires modification" for scores lower than 74.4; and "healthy diet" for scores equal to or greater than 74.4.

Additionally, the individual behavior of women in relation to BHEI-R was analyzed, identifying those who improved, worsened or remained in the same diet quality tertile, between T0T1 and between T1T2. Based on the classifications of each patient at T0T1 and at T1T2, they were grouped into five clusters of diet quality. These clusters received codes, namely: Constant, individuals who maintained a constant curve, being at the same classification for total BHEI-R tertile at all three times; Ascending, individuals who showed an ascending curve, increasing the total score of BHEI-R, changing to a higher tertile at T1 and again at T2. Also, those individuals that remained constant from T0 to T1, but showed an ascending curve from T1 to T2, and vice versa, were included in this cluster; Decreasing, individuals who showed a descending curve, decreasing the total score of BHEI-R, changing to a lower tertile at T1 and again at T2. Still, those individuals that remained constant from T0 to T1, but showed a descending curve from T1 to T2, and vice versa, were included in this cluster; V, individuals who showed a descending curve from T0 to T1, but ascending from T1 to T2; and Inverted V, individuals who had an ascending curve from T0 to T1, but descending from T1 to T2.

### Statistical analyses

First, the Kolmogorov-Smirnov normality test was performed. From the behavior of the variables, parametric tests for variables with normal distribution, or non-parametric tests for variables without normal distribution were performed. To analyze the diet quality, anthropometric measurements, nutritional status and nutrient intake during treatment (T0, T1 and T2), repeated measures ANOVA with post-hoc Tukey test were used; as well as Friedman, with post-hoc Dunn test. Moreover, a correlation of anthropometric variables with BHEI-R scores at the three times (T0, T1 and T2) was performed using Spearman or Pearson correlation tests. Confidence intervals (CI) of 0.95 and p values < 0.05 were considered. Statistical analyses were carried out using GraphPad Prism software, version 5.0.

The linear regression necessary for the adjustment of energy by the residual method was performed using the SPSS software, version 15.0 for Windows (SPSS Inc., Chicago, Illinois, USA).

## Results

**Fig 1:**
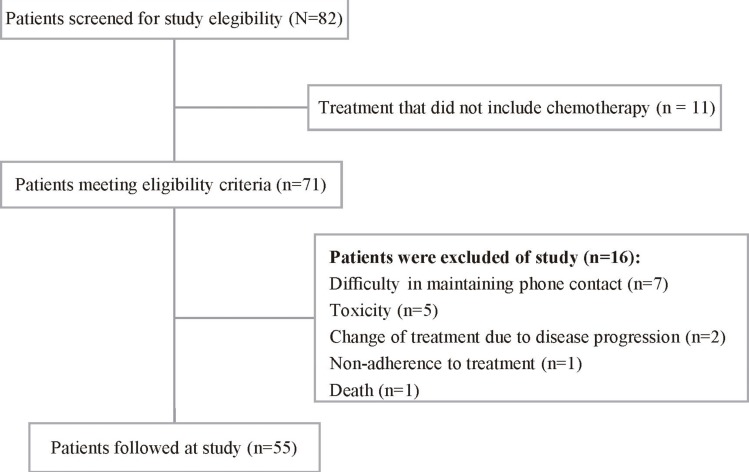
Diagram reporting the numbers of individuals at each stage of the study. Diagram reporting numbers of women with breast cancer screened, approached and recruited during the study at a university hospital in the city of Uberlandia, Minas Gerais, Brazil, 2014–2015 (n = 55).

The study included 55 women with a mean age of 51.5 ± 10.1 years. Fig 1 report numbers of women screened, approached and recruited at study.

Concerning clinical and hormonal characteristics, 61,8% (n = 34) were postmenopausal women; 96.4% (n = 53) had invasive ductal carcinoma; 47.3% (n = 26) were at clinical stage II; and 58.2% (n = 32) had moderately differentiated tumors. As the molecular subtype, the greater percentage of patients (41.8%, n = 23) were classified as luminal B, followed by 25.5% (n = 14) as luminal A. Among the patients who have undergone surgical procedures, 43.6% (n = 24) underwent breast-conserving surgery and 14.6% (n = 8) had mastectomy (Table 1).

**Table 1:** Clinical, hormonal and therapeutic characteristics (n = 55).

Variable	n	%
**Tumor subtype**		
Invasive Ductal	53	96,4
Invasive Lobular	2	3,6
**Clinical Stage**		
I	11	20,0
II	26	47,3
III	14	25,5
IV	1	1,8
NR	3	5,5
**Tumor Grade**		
G1	7	12,7
G2	32	58,2
G3	12	21,8
NR	4	7,3
**Molecular Subtypes**		
Luminal A	14	25,5
Luminal B	23	41,8
HER2-enriched	7	12,7
Triple Negative	11	20,0
**Menopausal Status**		
Premenopausal	21	38,2
Postmenopausal	34	61,8
**Surgery**		
Breast-conserving surgery	24	43,6
Mastectomy	8	14,6
No surgery	23	41,8
**Chemotherapy**		
Adjuvante	32	58,2
Neoadjuvante	23	41,8
**Chemotherapy Regimen**		
TAC	33	60,0
DC + Paclitaxel	8	14,6
FAC	9	16,4
CMF	5	9,1

NR, not reported; G1, well-differentiated tumor (low grade); G2, moderately differentiated tumor (intermediate grade); G3, poorly differentiated tumor (high grade); TAC, Docetaxel, Doxorubicin, and Cyclophosphamide; DC + Paclitaxel, Doxorubicin and Cyclophosphamide followed by Paclitaxel; FAC, Cyclophosphamide, Doxorubicin, and 5-Fluorouracil; CMF, Cyclophosphamide, Methotrexate, and 5-Fluorouracil.

The percentage of patients underwent adjuvant chemotherapy was 58.2% (n = 32) and 41.8% (n = 23) underwent neoadjuvant chemotherapy. The majority (60%, n = 33) were treated with TAC regimen (doxorubicin 60 mg/m^2^, i.v., day 1 plus cyclophosphamide 600 mg/m^2^, i.v., day 1, every 21 days, 4 cycles) followed by docetaxel (75–100 mg/m^2^, i.v., day 1, every 21 days, 4 cycles) (Table 1).

**Fig 2:**
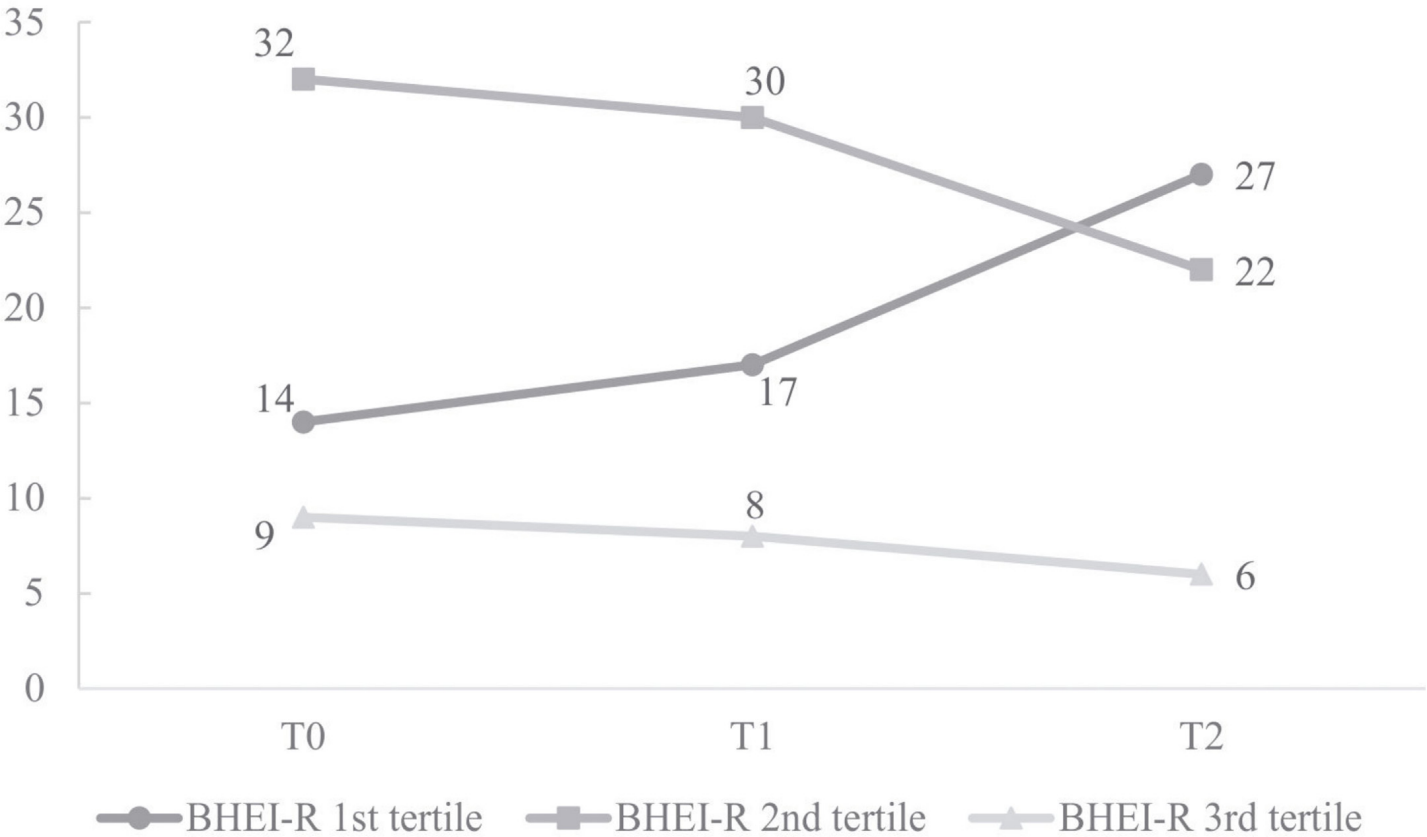
Distribution of women with breast cancer in tertiles of the BHEI-R (n = 55). Distribution of women with breast cancer in tertiles of the Brazilian Healthy Eating Index Revised (BHEI-R) at the start of chemotherapy (T0), intermediate chemotherapy (T1) and after chemotherapy (T2) in a university hospital in the city of Uberlandia, Minas Gerais, Brazil, 2014–2015 (n = 55). 1^st^ tertile, Inadequate diet; 2^nd^ tertile, Diet requires modification; 3^rd^ tertile, Healthy diet.

Regarding the BHEI-R analysis, the majority of women presented a "diet requires modification", both at T0 (58.2%, n = 32) and at T1 (54.5%, n = 30). However, at T2, the greater percentage of women (49.1%, n = 27) were classified with an "inadequate diet" (Fig 2).

**Fig 3:**
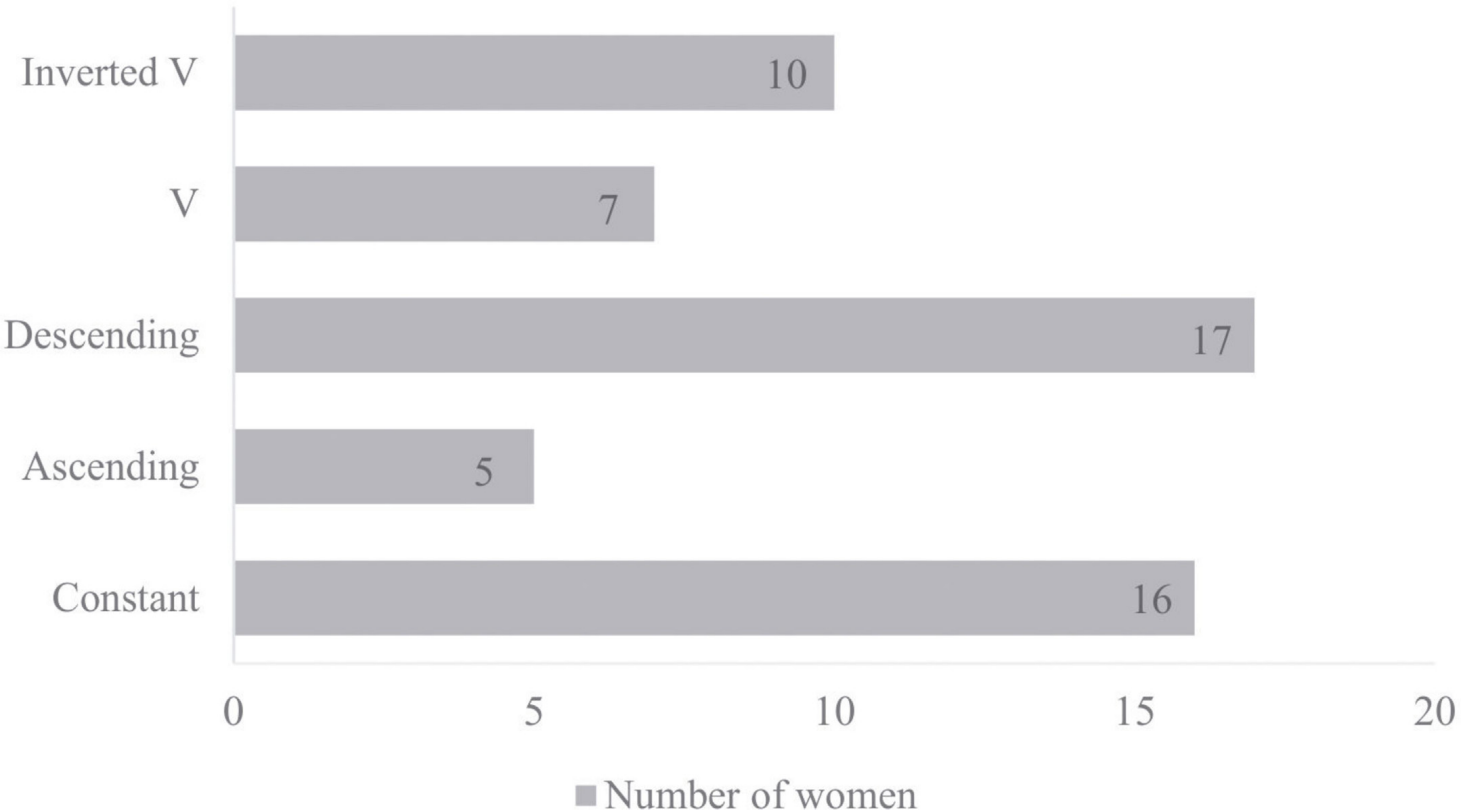
Distribution of women with breast cancer, according to diet quality clusters evaluated by the BHEI-R (n = 55). Distribution of women with breast cancer, according to diet quality clusters evaluated by the Brazilian Healthy Eating Index Revised (BHEI-R), during chemotherapy in a university hospital in the city of Uberlandia, Minas Gerais, Brazil, 2014–2015 (n = 55). Clusters: Inverted V, Patients who improved diet quality at T1 and worsened at T2; V, Patients who worsened the diet quality at T1 and improved at T2; Descending, Patients who worsened the diet quality at T1 and again at T2; Ascending, Patients who improved the quality of diet at T1 and again at T2; Constant, Patients who maintained the diet quality at all three times.

Analysis of the individual behavior of women in relation to BHEI-R during treatment found that the diet quality clusters with more representative percentages were Descending (30.9%, n = 17) and Constant (29.1%, n = 16) (Fig 3).

The evaluation of the total BHEI-R in the beginning (T0, mean = 68.2) and at the end of chemotherapy (T2, mean = 65.4), did not show statistically significant difference (p = 0.065, Table 2). When the analysis for the BHEI-R groups was performed, the scores for the Total Fruits, and Dark Green and Orange Vegetables and Legumes reduced with a statistically significant difference during treatment (means T0 = 3.8, T1 = 3.5, T2 = 3.1, p = 0.043; means T0 = 3.5, T1 = 3.1, T2 = 3.1, p = 0.026, respectively, Table 2). However, the post-hoc test was not able to detect this difference for Total Fruits, whereas for Dark Green and Orange Vegetables and Legumes the difference occurred between times T0 and T1.

**Table 2:** Scores of the total Brazilian Healthy Eating Index Revised (BHEI-R) and its components (n = 55).

BHEI-R Group (min.–max. Index scores)	T0		T1		T2		p
	Mean ± SD	Median (p25 –p75)	Mean ± SD	Median (p25 –p75)	Mean ± SD	Median (p25 –p75)	
Total Grains (0–5)	4.5 ± 0.7	4.8 (4.2–5.0) ^a^	4.6 ± 0.6	4.8 (4.4–5.0) ^a^	4.6 ± 0.5	4.9 (4.3–5.0) ^a^	0.223ᶷ
Whole Grains (0–5)	0.7 ± 1.1	0.0 (0.0–0.9) ^a^	0.7 ± 0.9	0.3 (0.0–1.4) ^a^	0.8 ± 1.4	0.0 (0.0–1.0) ^a^	0.250ᶷ
Total Fruits (0–5)	3.8 ± 1.2	4.1 (2.9–5.0) ^a^	3.5 ± 1.5	4.1 (2.6–4.9) ^a^	3.1 ± 1.6	3.3 (1.8–4.6) ^a^	***0*.*043***ᶷ
Whole Fruits (0–5)	3.6 ± 1.3	3.3 (2.9–5.0) ^a^	3.4 ± 1.6	3.3 (2.7–5.0) ^a^	3.1 ± 1.7	3.3 (1.7–5.0) ^a^	0.157ᶷ
Total Vegetables (0–5)	4.2 ± 0.9	4.4 (3.6–5.0) ^a^	3.9 ± 1.1	4.3 (3.3–4.9) ^a^	3.9 ± 1.2	4.1 (3.1–5.0) ^a^	0.349ᶷ
Dark Green and Orange Vegetables and Legumes (0–5)	3.5 ± 1.4	3.7 (2.6–4.9) ^a^	3.1 ± 1.4	3.0 (1.8–4.3) ^b^	3.1 ± 1.5	3.0 (2.0–4.4) ^a,b^	***0*.*026***ᶷ
Milk and Dairy Products (0–10)	4.5 ± 2.3	4.9 (2.5–6.4) ^a^	4.6 ± 2.7	4.4 (2.5–6.7) ^a^	4.4 ± 2.6	4.4 (2.1–6.1) ^a^	0.352ᶷ
Meat, Eggs and Beans (0–10)	7.6 ± 1.6 ^a^	7.9 (6.9–8.7)	7.4 ± 1.7 ^a^	7.6 (6.4–8.7)	7.6 ± 1.9 ^a^	8.1 (6.6–9.1)	0.708ᶿ
Oils (0–10)	9.8 ± 0.7	10.0 (10.0–10.0) ^a^	9.8 ± 0.8	10.0 (10.0–10.0) ^a^	9.9 ± 0.8	10.0 (10.0–10.0) ^a^	0.905ᶷ
Saturated Fat (0–10)	7.5 ± 1.7	7.9 (6.1–8.8) ^a^	7.2 ± 1.9	7.3 (6.1–8.8) ^a^	7.1 ± 2.3	7.6 (5.1–9.1) ^a^	0.458ᶷ
Sodium (0–10)	5.2 ± 1.6 ^a^	5.4 (4.0–6.6)	5.5 ± 1.9 ^a^	5.2 (4.4–6.8)	5.4 ± 1.8 ^a^	5.3 (4.1–6.5)	0.574ᶿ
Calories from SoFAAS^1^ (0–20)	13.3 ± 3.9 ^a^	14.1 (10.5–15.9)	12.5 ± 4.0 ^a^	13.2 (9.9–15.9)	12.5 ± 4.3 ^a^	13.1 (9.3–15.4)	0.375ᶿ
Total BHEI-R (0–100)	68.2 ± 7.1 ^a^	68.6 (62.7–72.3)	66.2 ± 8.2 ^a^	67.1 (61.7–71.1)	65.4 ± 8.1 ^a^	64.1 (60.1–72.2)	0.065ᶿ

T0, initial chemotherapy; T1, intermediate chemotherapy; T2, after chemotherapy; SD, Standard Deviation

^1^Calories from solid fats, alcohol and added sugars; Means/ medians horizontally followed by different letters differ statistically as post-hoc test at 5% probability

ᶿRepeated Measures ANOVA + Tukey Test

ᶷFriedman + Dunn Test.

In Table 3, are available the results of the nutritional status. Weight and BMI increased significantly (p = 0.008 and p = 0.009, respectively), indicating that these women had worsened their nutritional status, when compared at baseline (T0, weight, mean = 70.9Kg; BMI, mean = 28.4kg/m^2^) and after the end of treatment (T2, weight, mean = 71.8Kg; BMI, mean = 28.7kg/m^2^) (Table 4). WC also increased with statistically significant difference (p = 0.03) from the baseline (T0, mean = 90.8cm) to at the end of treatment (T2, mean = 91.1cm), but it was not detected in post-hoc test (Table 4). Furthermore, the correlations of BMI, WHR and WHTR after the end of chemotherapy (T2) were negative and statistically significant in relation to the total BHEI-R score, indicating that the worse the diet quality, the worse the nutritional status of patients (Table 4). The evaluation of the nutritional status of these women also indicated that the majority (T0 and T1, 56.3%, n = 31; T2, 54.5%, n = 30) were overweight at all three times.

**Table 3:** Classification of Body Mass Index, Waist Circumference, Waist-Hip Ratio and Waist-to-Height Ratio (n = 55).

Variable	Age group	T0		T1		T2	
Body Mass Index		n	%	n	%	n	%
Underweight	29–59	1	1.8	1	1.8	0	0.0
	60–66	2	3.6	2	3.6	2	3.6
Normal range	29–59	12	21.8	12	21.8	13	23.6
	60–66	9	16.4	9	16.4	10	18.2
**Overweight:**							
Preobese	29–59	9	16.4	9	16.4	10	18.2
	60–66	7	12.7	7	12.7	6	10.9
Obese class I	29–59	6	10.9	7	12.7	6	10.9
Obese class II	29–59	6	10.9	5	9.1	5	9.1
Obese class III	29–59	3	5.5	3	5.5	3	5.5
**Waist Circumference**							
Below the risk of metabolic complication (<80cm)	29–66	15	27.3	16	29.1	12	21.8
Increased risk of metabolic complication (≥80cm)	29–66	14	25.5	10	18.2	16	29.1
Substantially increased risk of metabolic complication (≥88cm)	29–66	26	47.3	29	52.7	27	49.1
**Waist-Hip Ratio**							
Do not have abdominal fat accumulation (≤0.85)	29–66	29	52.7	35	63.6	32	58.2
Abdominal fat accumulation (>0.85)	29–66	26	47.3	20	36.4	23	41.8
**Waist-to-Height Ratio**							
Do not have excess abdominal fat and metabolic risk (<0.5)	29–66	29	52.7	29	52.7	29	52.7
Excess abdominal fat and metabolic risk (≥0.5)	29–66	26	47.3	26	47.3	26	47.3

T0, initial chemotherapy; T1, intermediate chemotherapy; T2, after chemotherapy; BMI, Body Mass Index for adults [32] and seniors [42]; Waist Circumference [32]; Waist-Hip Ratio [32]; Waist-to-Height Ratio [33].

**Table 4:** Mean ± Standard Deviation of anthropometric variables and correlation of these variables with the Brazilian Healthy Eating Index Revised (BHEI-R) scores (n = 55).

Variable	T1		T2		T3		p	BHEI-R		
	Mean ± SD	Median (p25 –p75)	Mean ± SD	Median (p25 –p75)	Mean ± SD	Median (p25 –p75)		T0	T1	T2
**Weight (Kg)**	70.9 ± 16.4	66.1 (58.5–84.1) ª	71.4 ± 16.6	67.2 (58.6–83.9) ^a,b^	71.8 ± 16.8	66.4 (58.5–83.1) ᵇ	***0*.*008***ᶷ	-0.116^†^	-0.128^†^	-0.136^†^
**BMI (Kg/m**^**2**^**)**	28.4 ± 6.4	26.4 (23.5–33.7) ª	28.6 ± 6.5	26.3 (23.8–33.6) ^a,b^	28.7 ± 6.5	26.5 (23.9–33.3) ᵇ	***0*.*009***ᶷ	-0.080^†^	-0.1907^†^	***-0*.*254***^†^ *
**WC (cm)**	90.8 ± 15.7	86.5 (78.5–105.0) ª	91.2 ± 15.5	88.0 (79.0–103.0) ^a^	91.1 ± 15.3	87.0 (80.0–103.5) ª	***0*.*030***ᶷ	-0.119^†^	-0.077^***‡***^	-0.205^***‡***^
**WHR**	0.9 ± 0.1 ª	0.8 (0.8–0.9)	0.9 ± 0.1 ª	0.8 (0.8–0.9)	0.9 ± 0.1 ª	0.9 (0.8–0.9)	0.221ᶿ	-0.129^‡^	-0.028^***‡***^	***-0*.*325***^***‡***^ **
**WHTR**	0.6 ± 0.1	0.6 (0.5–0.7) ª	0.6 ± 0.1	0.6 (0.5–0.7) ^a^	0.6 ± 0.1	0.6 (0.5–0.7) ª	0.761ᶷ	-0.107^†^	-0.174^†^	***-0*.*272***^†^ ***

T0, initial chemotherapy; T1, intermediate chemotherapy; T2, after chemotherapy; BMI, Body Mass Index; SD, Standard Deviation; WC, Waist Circumference; WHR, Waist-Hip Ratio; WHTR, Waist-to-Height Ratio; Means/ medians horizontally followed by different letters differ statistically as post-hoc test at 5% probability

ᶿRepeated Measures ANOVA + Tukey Test

ᶷFriedman + Dunn Test

^†^Spearman correlation

^‡^Pearson correlation

*p = 0.03

**p = 0.008

***p = 0.022

Chemotherapy interferes in the diet of patients in quantitative terms having a negative impact on the micro and macronutrient intake over time as shown in Tables 5–7. Nutrients and energy had a modified consumption, with the exception of calcium, saturated fat, niacin and riboflavin, which did not change significantly during treatment. However, even with these nutrients not having experienced a significant change in intake, a high prevalence of inadequacy for calcium (100%), niacin and riboflavin (over 60% at T2) was observed.

**Table 5:** Food consumption disattenuated and adjusted for the residual method for the total energy (n = 55).

Energy and Nutrients	T0		T1		T2		p
	Mean ± SD	Median (p25 –p75)	Mean ± SD	Median (p25 –p75)	Mean ± SD	Median (p25 –p75)	
Energy (kcal) ^**I**^	1373.0 ± 257,6 ^a^	1377.7 (1217.2–1537.2)	1264.4 ± 333.1 ^b^	1243.1 (1031.3–1472.1)	1282.6 ± 265.9 ^b^	1224.6 (1094.5–1423.9)	***0*.*011*** ᶿ
Energy (kJ) ^**I**^	5744.6 ± 1077.8 ^a^	5764.3 (5092.8–6431.6)	5290.2 ± 1393.7 ^b^	5201.1 (4315.0–6159.3)	5366.4 ± 1112.5 ^b^	5123.7 (4579.4–5957.6)	***0*.*011*** ᶿ
Calcium (mg)	371.0 ± 69.6 ^a^	372,0 (322.5–414.7)	356.1 ± 112.7 ^a^	340.9 (289.6–445.8)	365.6 ± 107.1 ^a^	362.7 (288.0–430.7)	0.613 ᶿ
Carbohydrate (g)	184.5 ± 13.5	184.6 (174.3–193.9) ^a^	163.0 ± 13.0	164.9 (157.6–170.8) ^b^	164.9 ± 15.3	163.9 (156.0–175.3) ^b^	***<0*.*0001*** ᶷ
Cholesterol (mg)	170.7 ± 40.8	163.6 (152.2–187.8) ^a^	191.9 ± 37.6	191.8 (169.0–208.6) ^b^	185.9 ± 35.9	182.8 (163.8–207.2) ^a,b^	***0*.*002*** ᶷ
Iron (mg)	5.3 ± 1.1	5.2 (4.7–5.9) ^a^	4.5 ± 0.9	4.4 (3.9–5.0) ^b^	4.8 ± 1.2	4.6 (3.9–5.4) ^b^	***<0*.*0001*** ᶷ
Fiber (g)	17.2 ± 3.2 ^a^	17.1 (15.0–19.2)	14.5 ± 2.3 ^b^	14.3 (13.0–16.3)	14.0 ± 3.5 ^b^	13.8 (11.1–15.9)	***<0*.*0001*** ᶿ
Phosphorus (mg)	734.3 ± 90.4 ^a^	726.6 (669.4–787.7)	674.4 ± 96.3 ^b^	680.0 (560.0–733.0)	665.9 ± 77.3 ^b^	664.9 (610.4–701.8)	***<0*.*0001*** ᶿ
Monounsaturated fat (g)	12.5 ± 1.5 ^a^	12.5 (11.5–13.6)	11.5 ± 1.5 ^b^	11.5 (10.4–12.5)	11.6 ± 1.5 ^b^	11.6 (10.6–12.4)	***0*.*001*** ᶿ
Polyunsaturated fat (g)	12.8 ± 2.0 ^a^	12.7 (11.4–14.0)	11.1 ± 1.4 ^b^	11.0 (10.2–11.9)	11.2 ± 2.1 ^b^	11.2 (9.8–12.3)	***<0*.*0001*** ᶿ
Saturated fat (g)	13.6 ± 1.5 ^a^	13.5 (12.9–14.5)	13.1 ± 2.1 ^a^	13.2 (11.9–14.4)	13.5 ± 2.1 ^a^	13.2 (11.9–14.6)	0.240 ᶿ
Lipids (g)	44.6 ± 4.6 ^a^	45.2 (41.3–48.1)	42,3 ± 4.1 ^b^	41.6 (39.0–45.0)	42.6 ± 4.3 ^b^	42.1 (39.3–45.9)	***0*.*005*** ᶿ
Magnesium (mg)	174.1 ± 28.9	167.6 (156.6–187.3) ^a^	152.1 ± 25.8	144.8 (136.7–171.0) ^b^	152.3 ± 26.9	149.0 (136.5–167.8) ^b^	***<0*.*0001*** ᶷ
Manganese (mg)	2.1 ± 0.4	2.1 (1.8–2.3) ^a^	1.7 ± 0.4	1.7 (1.5–2.0) ^b^	1.5 ± 0.4	1.5 (1.2–1.6) ^c^	***<0*.*0001*** ᶷ
Niacin (mg)	10.7 ± 1.8	10.7 (9.1–11.4) ^a^	11.8 ± 3.1	11.3 (9.3–13.8) ^a^	10.7 ± 0.9	10.5 (10.0–11.2) ^a^	0.330 ᶷ
Potassium (mg)	3052.2 ± 369.5	2981.1 (2803.9–3319.4) ^a^	2528.1 ± 373.6	2452.7 (2245.4–2772.7) ^b^	2534.2 ± 369.0	2477.0 (2312.0–2780.3) ^b^	***<0*.*0001*** ᶷ
Protein (g)	55.9 ± 5.2 ^a^	55.4 (52.2–58.7)	51.1 ± 6.7 ^b^	50.3 (46.6–54.3)	53.2 ± 5.8 ^b^	52.8 (48.5–57.4)	***<0*.*0001*** ᶿ
Riboflavin (mg)	0.7 ± 0.2	0.7 (0.5–0.8) ^a^	0.7 ± 0.3	0.7 (0.5–0.9) ^a^	0.8 ± 0.3	0.7 (0.5–0.9) ^a^	0.959 ᶷ
Sodium (mg)	1800.5 ± 123.6 ^a^	1796.5 (1706.9–1885.5)	1554.3 ± 224.7 ^b^	1601.6 (1376.5–1690.4)	1645.0 ± 127.2 ^c^	1646.0 (1575.9–1695.7)	***<0*.*0001*** ᶿ
Thiamine (mg)	1.0 ± 0.4	0.9 (0.8–1.2) ^a^	1.2 ± 0.7	1.0 (0.9–1.5) ^b^	1.0 ± 0.2	1.0 (0.9–1.1) ^a,b^	***0*.*036*** ᶷ
Vitamin B6 (mg)	0.5 ± 0.1 ^a^	0.5 (0.4–0.5)	0.5 ± 0.1 ^a^	0.5 (0.4–0.5)	0.5 ± 0.1 ^b^	0.5 (0.5–0.6)	***<0*.*0001*** ᶿ
Vitamin C (mg)	183.2 ± 76.4	171.2 (131.2–240.1) ^a^	157.2 ± 73.1	151.6 (98.5–196.5) ^b^	125.2 ± 69.4	106.9 (81.6–162.9) ^b^	***0*.*0001*** ᶷ
Zinc (mg)	7.8 ± 0.7	7.7 (7.4–8.3) ^a^	5.7 ± 1.3	5.5 (4.7–6.6) ^b^	6.5 ± 1.4	6.1 (5.3–7.8) ^b^	***<0*.*0001*** ᶷ

T0, initial chemotherapy; T1, intermediate chemotherapy; T2, after chemotherapy; Disattenuated, Adjusted for intra-individual variability, proposed by [37]; Adjusted for energy, Adjusted for total energy consumption by the residual method, proposed by [38]; SD, Standard Deviation; ^I^Values only disattenuated. Means/ medians horizontally followed by different letters differ statistically as post-hoc test at 5% probability

ᶿRepeated Measures ANOVA + Tukey Test

ᶷFriedman + Dunn Test.

**Table 6:** Prevalence of nutrient intake inadequacy using the EAR method as the cut-off point, and comparison of intake with the AI (n = 55).

Nutrient	Age group (years)	DRI	Prevalence of Inadequacy^1^ (%)		
			T0	T1	T2
		**EAR**			
Calcium (mg)	19–50	800	100	100	100
	51–70	1000	100	100	100
Iron (mg) ^2^	19–50	8.1	100	100	99.7
	51–70	5	33.7	66.6	53.6
Phosphorus (mg)	19–70	580	4.5	16.4	13.4
Magnesium (mg)^3^	31–70	265	99.9	100	100
Niacin (mg)	19–70	11	56.4	39.7	63.7
Riboflavin (mg)	19–70	0.9	80.2	72.2	65.5
Thiamine (mg)	19–70	0.9	38.2	30.5	28.4
Vitamin B6 (mg)	19–50	1.1	100	100	100
	51–70	1.3	100	100	100
Vitamin C (mg)	19–70	60	5.4	9.3	17.6
Zinc (mg)	19–70	6.8	7.9	79.1	57.5
		**AI**	**Intake comparison with AI**		
Fiber (g)	19–50	25	Below	Below	Below
	51–70	21	Below	Below	Below
Manganese (mg)	19–70	1.8	Above*	Below	Below
Potassium (mg)	19–70	4700	Below	Below	Below
Sodium (mg)	19–50	1500	Above**	Above**	Above**
	51–70	1300	Above**	Above**	Above**

T0, initial chemotherapy; T1, intermediate chemotherapy; T2, after chemotherapy; DRI, Dietary Reference Intake [40]; EAR, Estimated Average Requirement; AI, Adequate Intake

^1^Cannot be calculated for nutrients that do not have EAR

^2^Only postmenopausal women were included in prevalence analysis

^3^A patient under the age of 31 years was excluded from prevalence analysis

*Below UL (Tolerable Upper Intake Level) of 11mg [40].

**Below UL of 2300mg [40].

**Table 7:** Mean ± Standard Deviation of cholesterol intake, and percentage of adequacy of macronutrients in relation to nutritional recommendations (n = 55).

Nutrients	Recommendation	T0	T1	T2
Cholesterol (mg)	- ^I^	170.7 ± 40.8	191.9 ± 37.6	185.9 ± 35.9
Carbohydrates (%)	45–65% ^II^	53.8	51.6	51.4
Proteins (%)	10–35% ^II^	16.3	16.2	16.6
Lipids (%)	20–35% ^II^	29.2	30.1	29.9
Monounsaturated fat (%)	15–20% ^I^	8.2	8.2	8.1
Polyunsaturated fat (%)	6–11% ^I^	8.4	7.9	8.0
Saturated fat (%)	< 10% ^I^	8.9	9.3	9.5

T0, initial chemotherapy; T1, intermediate chemotherapy; T2, after chemotherapy

^I^ [41]

^II^AMDR, Acceptable Macronutrient Distribution Range [40]. The cholesterol intake should be minimized while consuming a nutritionally adequate diet.

Cholesterol and thiamine intake increased during treatment, with a significant increase from T0 to T1 as well as for vitamin B6, between T0 and T2 (Table 5). As for B6 and thiamine vitamins, even though there was an intake increase during the treatment, it was not enough to ensure adequacy in relation to the EAR, and inadequacy prevalence of 28.4% and 100%, respectively, were identified after the end of chemotherapy (Table 6).

The other nutrients and energy had a significant reduction in consumption, exacerbating the inadequacy prevalence of iron, phosphorus, magnesium, vitamin C and zinc. Compared with AI, fiber, manganese and potassium presented an intake below the recommended levels, but quantitative conclusions cannot be made, since intakes below the AI will still be suitable for a group of individuals. Sodium intake (T0, T1 and T2) and manganese (T0) remained above AI and below UL, which is certainly an adequate intake (Table 6).

Regarding macronutrients, despite the significant reduction in consumption during treatment (Table 5), they remained within recommendations [40, 41], except for the consumption of monounsaturated fat below recommendations (Table 7).

## Discussion

Changes in the diet of women with breast cancer were observed during chemotherapy, even though these modifications have been better evidenced in quantitative rather than qualitative terms. Calcium, saturated fat, niacina and riboflavina were the only nutrients that did not show significant consumption change. Cholesterol, thiamine and vitamin B6 showed a significant increase, while the other thirteen nutrients and energy significantly reduced, during treatment. Furthermore, high prevalence of inadequacy, up to 100%, were identified for calcium, iron, phosphorus, magnesium, niacina, riboflavina, thiamin, vitamin B6, vitamin C and zinc.

The majority of women had a “diet requires modification’, both at the beginning and during treatment, and a "inadequate diet" at the end of the CT. The Total Fruit consumption as well as the Dark Green and Orange Vegetable and Legume consumption decreased significantly during treatment. The most representative clusters of diet quality were Decreasing and Constant. The greater percentage of patients began chemotherapy with excess body weight, which was aggravated due to the statistically significant increase in weight, BMI and WC during CT. Still, correlations were observed between poor diet quality and higher values for BMI, WHR and WHTR. Few studies have used prospective longitudinal model to evaluate the triad food consumption, chemotherapy and breast cancer, highlighting the importance of the results presented.

As the treatment affects not only tumor cells, the occurrence of side effects is known, such as dysgeusia, reduced appetite, nausea, oral mucositis, and dry mouth among others that can affect dietary intake and nutritional outcomes [43].

In Brazil, a study that evaluated the diet quality of patients with breast cancer found no statistically significant difference between BHEI-R’ average scores at the basal period and during treatment (p = 0.907) [44]. However, while analyzing BHEI-R’ components were observed that the total Fruit consumption decreased significantly (p = 0.002) and the sodium intake increased significantly (p = 0.029) [44].

In this study, the significantly reduced consumption of Total Fruits and Dark Green and Orange Vegetables and Legumes, during the treatment, probably contributed in worsening the supply of vitamins and minerals. A meta-analysis found that a high intake of fruits and the combination of fruits and vegetables, but not of vegetables alone, is associated with a moderate reduction in the risk of breast cancer [45]. However, the evidence of the relationship between consumption of these foods and reduction of the disease risk is still limited [18, 46]. Nevertheless, it is widely agreed that the consumption of fiber and soy, as well as a low consumption of total fats, especially saturated fats, are factors associated with improved survival [18, 47], besides being positively related to cardiovascular health [48].

It is noteworthy that the Milk and Dairy Products consumption has not reduced statistically during treatment, but this consumption was low at all times, which may have contributed to the high prevalence of inadequacy for calcium, 100%, observed at three times. Study reported trend, although not statistically significant (p = 0.09) between higher calcium intake and reduced risk of death from breast cancer [49].

Another aspect to consider is that nutrients, such as vitamin B6, magnesium, riboflavin, thiamine, zinc and niacin, which had a high prevalence of inadequacy in this study, have anti-inflammatory properties, thus their consumption has the potential to improve the anti-inflammatory cytokine profile and may reduce the risk of adverse health outcomes of these patients [50]. In contrast, a pro-inflammatory diet, characterized by high amounts of carbohydrates, proteins, total lipids, saturated fats, cholesterol, and *trans* fats, among others, seems to increase the risk of breast cancer, especially among women after menopause [51]. The high intake of saturated and *trans* fat is associated with an increased risk of death from any cause among women with breast cancer [49].

The study found that higher scores of Dark Green and Orange Vegetable and Legume were associated significantly with lower concentrations of C-reactive protein (CRP) [16]. Elevated CRP and serum amyloid A (SAA) were associated with reduced overall survival in women diagnosed with breast cancer, regardless of age, race, BMI and tumor stage [52]. Literature has presented evidence that a higher quality diet seems to be associated with lower levels of chronic inflammation and, consequently, better survival from the disease [16, 53, 54].

A study that evaluated the diet quality of patients with breast cancer, using the HEI-2005, found that those who had a higher quality diet showed an 60% lower risk of death from any cause and 88% lower risk of death from breast cancer, highlighting the positive influence of adopting better eating habits on the survival of these women [19]. Study suggests that a high quality diet after diagnosis does not significantly alter the risk of death from breast cancer, but still, the importance of healthy food choices are emphasized, as these women are at risk of death from other causes not related to cancer, but affected by diet [20].

There is evidence that common problems that affect women diagnosed with breast cancer, such as a recurrence of the disease, loss of bone density, cardiovascular disease, cognitive dysfunction, and peripheral neuropathy associated with chemotherapy can be minimized, even prevented, with the consumption of certain foods and nutrients [55]. A study reported an association between a higher dietary intake of eicosapentaenoic acid (EPA) and docosahexaenoic acid (DHA) and a 25% reduction in the recurrence of breast cancer, as well as a reduction in overall mortality [56]. Additionally, these nutrients have favorable effects on bone, and the central nervous and cardiovascular systems [55]. Another study found that the consumption of fruits and vegetables was positively associated with better cognitive performance, both for women with breast cancer and for the control group [57]. This outcome is consistent with other study that found that better post-diagnosis diet quality is directly associated with better scores of mental and physical functioning in breast cancer survivors [58].

When BHEI-R and anthropometric variables were correlated, it was found that, statistically, a worse diet quality is correlated with higher values for BMI, WHR and WHTR. Although the relationship between body weight and breast cancer survival is not conclusive, the evidence is strong between overweight or obesity and the risk for the occurrence of eight types of cancer, including breast cancer in postmenopausal women [18]. Furthermore, it is known that obesity compromises self-esteem [29] and predisposes the person to the risk of serious comorbidities, such as type 2 diabetes and cardiovascular disease (CVD) [17, 29], making women more prone to dying from breast cancer [59].The CVD are the second most common cause of mortality in women with breast cancer [55] and the most common cause among those with breast cancer and older than 65 years [55; 59]. Additionally, patients with breast cancer, especially elderly women, who were treated with adjuvante CT may be subject to an increased risk of CVD due to the possible association between this treatment and long-term cardiotoxicity [60]. Adoption of healthy dietary habits has proven effective in reducing the risk of such comorbidities [48].

A prospective cohort study found that HEI-2005 was not associated with death from breast cancer (p = 0.627), however it was found that the better post-diagnosis diet quality is associated with 26% lower risk of death from any cause (p = 0.043) and 42% lower risk of death from non-breast-cancer causes (p = 0.011) [22]. The better diet quality was associated with a reduced risk of all-cause of mortality among women with ER+ tumors (p = 0.0009), suggesting that the effects of diet quality may differ between tumor subtypes [22]. The quality of post-diagnosis diet can have a greater effect in promoting longevity for these women and can further contribute to cardiovascular health than in disease progression [22].

A reduction of the patients’ weight was expected, which did not occur, although reduced the caloric intake during chemotherapy. One of the limitations of this study negatively affects greater inferences regarding weight gain, such as not using bioimpedance to determine whether this increase in body weight was due to fat gain or water retention, since the treatment included the use of corticoids. However, studies show that weight gain is common among women treated with chemotherapy [12, 29, 61], also with a significant increase in the percentage of body fat [12, 18]. In addition, women with breast cancer may experience declining levels of physical activity after diagnosis, which also contributes to weight gain [62]. It is noteworthy that such changes in weight and also in diet cannot be expected for these women, causing concern and disappointment that could be prevented by appropriate guidance in the post-diagnostic phase [29].

Increased BMI favors the elevation of insulin and growth fator similar to insulin (IGF), and is associated significantly with an increased risk of breast cancer development and progression of disease [63]. Additionally, the higher the adiposity, the degree of insulin resistance and hyperinsulinemia, the lower the plasma concentration of adiponectin, an anti-inflammatory hormone with a protective effect against type 2 diabetes, CVD [64] and breast cancer [65].

Although this study suggests that chemotherapy has an impact on the overall quality of the diet of patients with breast cancer, this result was not statistically significant in this relatively small sample, although it has reached the sample calculation. Nevertheless, significant reductions in the consumption of certain food groups, micro and macronutrients, as well as changes in nutritional status could be confirmed.

We must also consider other possible limitations of this study. One of the prerequisites to implement the EAR method as the cut-off point is that the distribution of nutrients needs to be symmetrical (not necessarily normal). This method is not recommended for iron in women of childbearing age, whose needs have known asymmetrical distribution, due to menstrual losses. Therefore, this method was applied only to women that were in postmenopause (61.8%). Also, it is likely that the result of calculating the prevalence of inadequacy of this micronutrient is underestimated given that the wheat flour present in TACO is not be enriched with iron, being below the required in legislation and usual intake of Brazilians. Since 2004, the publication of Resolution RDC 344 [66] determined that manufacturers should enrich wheat flour and corn flour with iron (4.2mg/ 100g) and folic acid, but the TACO, even in its 4th edition of 2011, brings only 1mg of iron per 100g of wheat flour [35].

To evaluate the dietary intake a 24HR was used, since this method is influenced by the individual's ability to recall the food consumption accurately, which may have led to underestimation by memory bias, also inevitable due to the recent cancer diagnosis context. However, in order to minimize this limitation, the interviews were conducted by trained nutritionists, nine 24h dietary recalls were applied to each individual, and nutrients were disattenuated and adjusted for energy in order to reduce the intra-individual variability and to reflect the closest possible intake to reality of these individuals.

The intra-individual variability, usually heterogeneous among individuals, is due to both the failure to correctly report the amounts of food actually consumed and the individual’s day-to-day variability in food consumption [67]. The collection of three 24HR in each of the times allowed the setting of this variability, which is a strength of the study. The calculation of prevalence of inadequacy after correction of intra-individual variability and the adjustment of energy allow that the distribution of consumption only reflects the variation between individuals, preventing underestimation or overestimation the inadequacy of nutrients.

Changes in the perception of taste and smell of food can cause reduction of pleasure in eating [29]. Although some women tend to opt for a healthier diet after diagnosis, there are reports that they find comfort in consuming less healthy foods, using them as a reward for the strenuous and difficult period of chemotherapy [29]. Although this study has identified changes in eating behavior during treatment, the reasons remain to be further investigated. For this reason, it is suggested that a qualitative study aimed at elucidating possible factors which women consider responsible for this change be carried out.

## Conclusion

The results of this study show that women with breast cancer during chemotherapy are overweight and have changes in diet quality, with a significant reduction in the intake of total fruits and dark green and orange vegetables and legumes being observed. Furthermore, the decreased consumption of micro and macronutrients was alarming, and a high prevalence of inadequacy was identified during treatment.

Such results reinforce the importance of the monitoring and guiding of these women by health care professionals, regarding the measures to be adopted to ensure that dietary quality is maintained within a healthy standard, with an adequate supply of nutrients, avoiding a compromised nutritional status and contributing to better recovery, quality of life and a reduction in the risk of recurrence.
